# Functional Magnetic Resonance Imaging for Language Mapping in Temporal Lobe Epilepsy

**DOI:** 10.1155/2012/198183

**Published:** 2012-07-25

**Authors:** An Wang, Terry M. Peters, Sandrine de Ribaupierre, Seyed M. Mirsattari

**Affiliations:** ^1^Imaging Research Laboratories, Robarts Research Institute, London, ON, Canada N6A 5K8; ^2^Department of Medical Biophysics, Western University, London, ON, Canada N6A 5C1; ^3^Department of Medical Imaging, Western University, London, ON, Canada N6A 5A5; ^4^Biomedical Engineering Program, Western University, London, ON, Canada N6A 5B9; ^5^Department of Clinical Neurological Science, Western University, London, ON, Canada N6A 5A5; ^6^Department of Psychology, Western University, London, ON, Canada N6A 5A5

## Abstract

Functional magnetic resonance imaging (fMRI) is a noninvasive technique that is increasingly used to understand the cerebral cortical networks and organizations. In this paper, we describe the role of fMRI for mapping language networks in the presurgical workup of patients with medically intractable temporal lobe epilepsy (TLE). Studies comparing fMRI with the intracarotid sodium amobarbital (Wada) test and fMRI with intraoperative cortical stimulation mapping for language lateralization and/or localization in medically intractable TLE are discussed.

## 1. Introduction

Epilepsy is a common neurological disorder that affects about 0.5%–1% of the general population. Temporal lobe epilepsy (TLE) is the most common form of partial epilepsy [1]. About 20%–30% of the patients with TLE develop medication resistance and may benefit from a surgical treatment. For patients with medically refractory TLE, surgical excision of the affected temporal lobe is an alternative approach to the treatment of this disorder [2]. While anterior temporal lobectomy (ATL) has proven to be effective in the treatment of TLE patients resistant to medical therapy [3], successful surgical outcome depends not only on an accurate localization of the epileptogenic focus, but also on the ability to map and preserve the “eloquent cortex.” In particular, the lateralization and localization of language cortex are of paramount importance for ATL in the dominant hemisphere. As demonstrated by Haglund et al. [4], the distance from the language cortex to the resection boundary is the most important predictor of developing postoperative language deficits.

Currently, invasive tests such as the intracarotid amobarbital procedure (IAP, also called the Wada test) [5] and electrocortical stimulation mapping (ESM) [6], both of which present the patient with additional risks, are the common means for lateralization and localization of the functional regions in brain. Noninvasive imaging techniques may reduce the need for such procedures, and, in particular, functional magnetic resonance imaging (fMRI) is an alternative to replace these invasive procedures for surgical planning of the patients with TLE.

In the following section, we review the basics of fMRI, while Sections 3 and 4 review previous studies comparing fMRI to the Wada test for language lateralization and fMRI to ESM for language localization. In Section 5, we discuss the role of fMRI in studying brain plasticity for language function after temporal lobectomy (TLY).

## 2. Basics of fMRI

fMRI employs the blood-oxygenation-level-dependent (BOLD) [7] contrast mechanism as an indirect measure of underlying neuronal activity. When certain functional areas in brain are activated, there is an increase in local metabolism and oxygen consumption. Because of the neurovascular coupling between regional changes in brain metabolism and cerebral blood flow (CBF), the activated local areas in the brain experience a decrease in oxyhemoglobin and an increase in deoxyhemoglobin in the postcapillary vascular bed. Since hemoglobin has different magnetic properties depending on its state of oxygenation, being diamagnetic when oxygenated and paramagnetic when deoxygenated, these oxygen-related changes to the blood lead to local magnetic field inhomogeneities, which in turn result in detectable changes in the magnetic resonance signal measurable by MRI. 

To elicit the activated functional areas in brain using the BOLD signal effectively and robustly, most fMRI studies employ a block design, in which tasks are alternated to generate brain response in different states (in the simplest form, two states: rest versus activation). Event-related designs have also been employed in the last few years to examine language function and lateralization. In contrast to a block design, in which the conditions are alternated within a block (resting, task1, resting, task2) with each block having a fixed duration, in an event-related design, events of different types are randomly intermixed. Some studies have shown a more robust activation of language areas in event-related than in block design paradigms [8]. 

**Figure 1:**
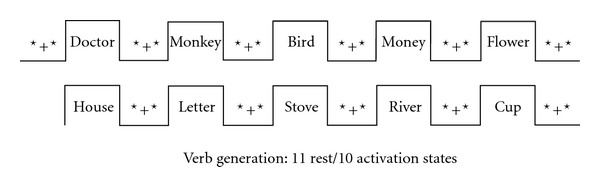
Schematic diagram of a verb generation task used at our centre.

Because the magnetic signal changes caused by the hemodynamic behaviour are usually very small, the selection and design of the experimental paradigm is of utmost importance. Various auditory and visual stimulation paradigms have been developed to examine the language functions, and since language tasks are most likely to activate not only language areas but also other functional regions, the experimental paradigm requires controlled tasks that activate nonlanguage cortex equally. A common example used for language study that can be performed easily in patients for lateralization purposes is the “verb generation” and “verbal fluency” task. In this type of design, participants are instructed to covertly generate action verbs for each noun (e.g., for the word “knife” one can think “to cut,” “to slice,” “to throw”). The resting phase involves participants looking at a cross in the center of the screen while not actively engaging in any language function. Alternatively, subjects may be presented with nonsense collections of letters to “washout” the nonlinguistic aspects of the task. This task provides relatively consistent activation of the anterior language areas (Figure 1). Another commonly used language task employed at our institution is a “sentence completion paradigm” (also called “sentence comprehension”). Participants are informed they will see a sentence on the screen, which they must covertly complete, such as “I CALLED THE …”, “WOMEN WEAR …”, “WE CLEANED …”, “I LOVED ….” The resting phase task involves participants looking at the instructional word “RELAX” projected onto the screen. Compared to the “verb generation” task, the “semantic decision-making” task demonstrates more widely distributed networks, including the anterior and posterior language areas. In this type of design, for example, the subjects are instructed to compare the samples in terms of their meaning and to select two out of three words that are “most alike.”

The most commonly used imaging sequence in fMRI studies is echo planar imaging (EPI), due to its fast acquisition time. However, the temporal resolution of such a sequence is in the order of several seconds, as it requires some time to produce detectable hemodynamic changes after stimulus onset and the spatial resolution is usually significantly lower than that of anatomical MRI. Another drawback is that EPI is sensitive to field inhomogeneities, leading to geometric distortion of the images in certain brain regions.

**Figure 2:**
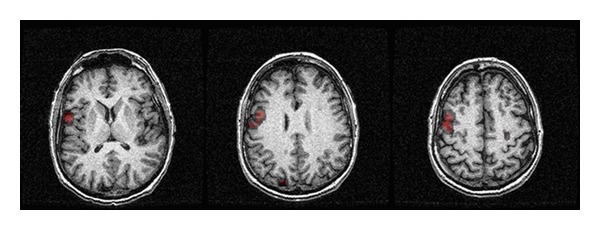
fMRI activation map overlaid on anatomical images.

Since the BOLD signal is extremely sensitive to motion, one of the common problems during any fMRI experiment is subject motion, which can compromise the entire experiment. Motion can range from gross head movement to the minimal brain motion associated with cardiac or respiratory cycles, a.k.a. the cardioballistic effect. Before any subsequent analysis can be performed, the individual images are commonly realigned and coregistered to minimize the motion effect. Next, the signal time course in each voxel of the images and the time course of different tasks are correlated using statistical modeling. Various statistical tests (e.g., “Student's  *t-*test”) can then be applied on a voxel-by-voxel basis to examine the probability that a particular voxel with an increased signal is associated with a particular functional state of the brain. The result of this process is a map of the voxels that show statistically significant changes associated with the brain function under investigation. This step is critical since the fMRI signal changes are usually very small (in the order of 0.5%–5%), leading to a high probability of false negative results. To better appreciate the anatomical location of the origin of the signal, these statistical maps are usually registered and fused to a high-resolution anatomical image (Figure 2).

As described above, fMRI analysis requires considerable mathematical, statistical, and image processing that is provided by a variety of free or commercial software packages, such as statistical parametric mapping (SPM) (http://www.fil.ion.ucl.ac.uk/), FSL (http://www.fmrib.ox.ac.uk/fsl/), AFNI (http://afni.nimh.nih.gov/afni), and Brain Voyager (http://www.brainvoyager.com/).

## 3. Role of fMRI in Lateralization of Language Functions

### 3.1. Language Lateralization Using the Wada Test

The Wada test consists of unilateral injection of sodium amobarbital into the internal carotid artery (ICA), which temporarily anaesthetizes the hemisphere ipsilateral to the injection site. While one hemisphere is anaesthetized, language and memory functions of the hemisphere contralateral to the injection site can be tested. To test cerebral dominance for language, the patient is asked to perform a number of tasks involving expressive and receptive language. For example, tasks involving counting numbers, naming the months of the year, or naming objects are often used to examine frontal language areas, while repetition, responding to verbal commands, and reading are employed to explore language functions served by the posterior brain regions. These protocols can be adapted or simplified for children or mentally challenged individuals, as long as they possess expressive language and are able to understand the instructions. The test is administered before the injection to provide a baseline measure and is repeated after the anaesthesia has taken effect. If the hemisphere dominant for speech is anaesthetized, the patient is temporarily rendered mute, which is not the case when the nondominant hemisphere is deactivated. When each hemisphere retains some language functions following unilateral injection, bilateral representation is confirmed.

**Figure 3:**
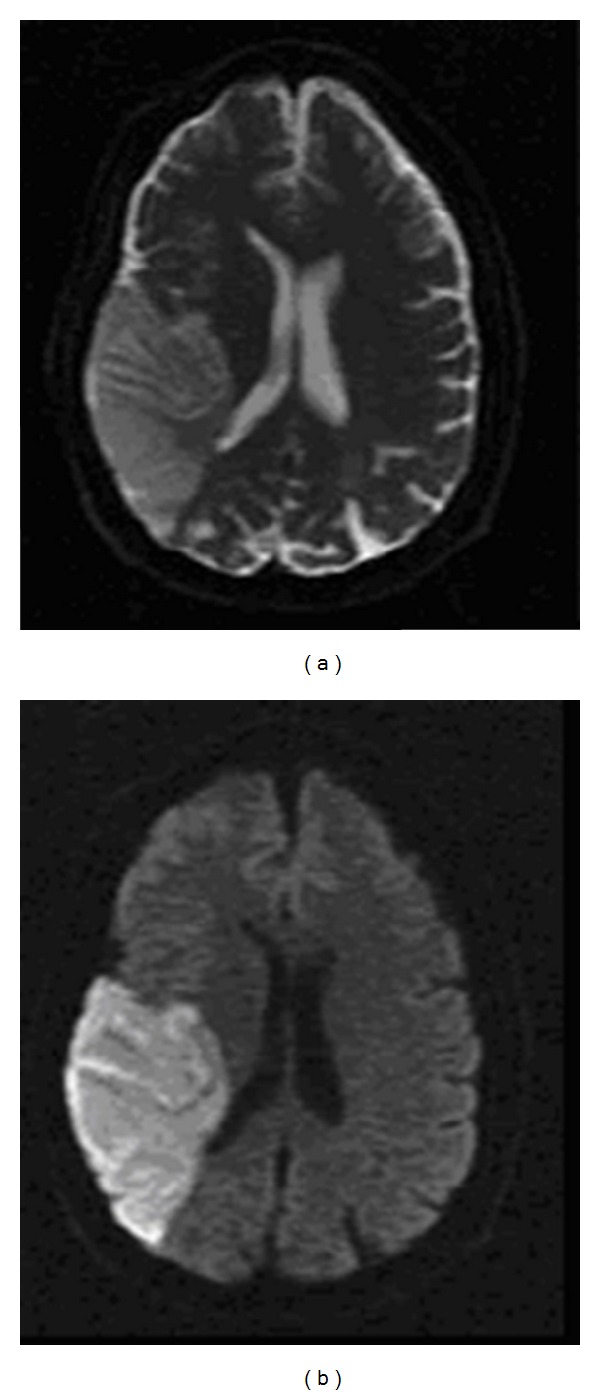
Axial T_2_ (a) and axial diffusion-weighted (b) images of a TLE patient who experienced a stroke in the right middle cerebral artery territory secondary to a traumatic dissection of the right internal carotid artery during the sodium amobarbital testing.

Although the Wada test has long been considered as the gold standard for preoperative language and memory testing, the method has several drawbacks, since it is highly invasive and quite uncomfortable for most patients, and there is a small risk of morbidity. Figure 3 shows axial T_2_ (a) and axial diffusion-weighted (b) images of a patient with medically intractable TLE of left temporal lobe origin who had experienced a right middle cerebral artery stroke secondary to a traumatic dissection of the right ICA during the Wada test. Another major drawback for the Wada test is that the procedure requires the patient to respond verbally, making it difficult to obtain reliable results from young children and mentally challenged patients. Finally, the Wada test only provides information about lateralization, but not localization of cognitive functions. These important limitations have led many epilepsy centers to seek alternative means to probe language and memory functions in patients prior to epilepsy surgery.

### 3.2. Concordance of Language Lateralization Using fMRI with the Wada Test

A number of studies have examined the utility of fMRI as compared to the Wada test for language lateralization in patients with various neurological conditions, with reports being related specifically to epilepsy and TLE. One of the first published studies to compare fMRI with the Wada test in patients with TLE was described by Desmond et al. [9], where the authors employed a semantic task during which the patients pressed a button when they recognised a word as being abstract as distinct from being concrete. The hemispheric language laterality index was calculated using the formula

(1) LI=(VL−VR)(VL+VR)×100,

where *V*_*L*_ and *V*_*R*_ are activation volumes for the left and right hemispheres, respectively. Desmond et al. found that the fMRI laterality indices were in agreement with the Wada test in all of their seven patients. Binder et al. [10] used a semantic decision task, in which the patients pressed a button if they heard a word that was both “animal native to the US” and “commonly used by humans.” The laterality indices were in concordance with the Wada test for all of their 22 patients with epilepsy. Following these two studies, Bahn et al. [11] studied four epilepsy patients with two relatively simple covert word generation tasks and found 100% concordance between fMRI and the Wada test in all their four patients with epilepsy. In a study conducted by Yetkin et al. [12], a group of 13 patients with medically intractable epilepsy were tested with a verbal fluency task. Good concordance between fMRI and the Wada test with the laterality correlation coefficient of 0.96 between the two techniques was reported except for one patient. Lehéricy et al. [13] studied ten patients with TLE using a panel of language paradigms including verbal fluency, story-listening, and sentence repetition tasks. They found that fMRI laterality indices were highly concordant with the Wada test in the frontal lobe, although this was not the case in the temporal lobe. Moreover, they found that a verbal fluency task was more effective than other tasks in achieving better correlation with the Wada test.

While the previous studies have shown promising concordance between fMRI and the Wada test, the patients in these studies had mainly unilateral dominant epilepsy. Rutten et al. [14] enrolled seven patients with unilateral and six with bilateral TLE to investigate the predictive power of fMRI for language lateralization. They concluded that using a combination of language tasks that included verb generation, verb fluency, picture naming, and sentence comprehension, they were able to better predict the lateralization of these patients. Another study by Sabbah et al. [15] also tried to investigate the effectiveness of fMRI in lateralization of atypical language in patients with epilepsy. By using two different semantic fluency paradigms, they reported that 19 out of 20 patients had concordant lateralization result from both fMRI and the Wada test. Comparing fMRI-based and Wada-based laterality quotients for speech in TLE patients, Benke et al. [16] found agreement in 89.3% of right TLE patients and in 72.5% of left TLE patients. However, while fMRI correctly detected atypical right hemisphere speech in all cases, it missed left hemispheric dominance of speech in 17.2% of patients with TLE. Furthermore, the method was less sensitive to bilateral speech representation.

While higher magnetic field strength is desirable for fMRI studies, this may not be necessary for language lateralization. For instance, Deblaere et al. [17] used a simple word generation paradigm in an 1.0 T MR scanner and showed that fMRI reliably lateralized the language function in a clinical setting. Although most of the previous studies focused on expressive speech, Gaillard et al. [18] conducted an fMRI study in which a combination of expressive and receptive language paradigms was employed. They reported that there was a complete agreement between the Wada and fMRI in 21 of 25 patients and that the use of multiple tasks increased the accuracy in determining hemispheric dominance for language function.

To date, the largest study on comparison between fMRI and the Wada test was conducted by Woermann et al. [19], who enrolled 100 patients with epilepsy, of whom 69 had TLE. They employed a single covert word generation language paradigm and derived the laterality based on visual inspection of fMRI statistical maps. Concordance of 91% was observed, and the discordance may have been the result of visually assessing often bilateral, although mainly asymmetric fMRI activations.

Generally, fMRI and the Wada test agree very well with each other. Because of the relative advantages of fMRI, more and more epilepsy centers are replacing the Wada test with fMRI for presurgical language mapping. However, we also note some divergent results from the previous studies, which can be attributed to several factors. First of all, the fMRI paradigms used to evaluate language lateralization vary considerably between studies. Most of the paradigms are capable of activating the left frontal lobe in healthy control subjects and patients with typical representations for language functions. In patients with atypical representations for language, the use of multiple language tasks increases the likelihood of accurate lateralization of the cerebral hemispheres for language functions. Other limitations of fMRI for language mapping include the variability in statistical thresholds employed to calculate the activated volumes, difficulties in determining the extent to which the right hemisphere participates in language processing in patients with bilateral representations for language networks, and because not all areas involved in a task may be activated by a particular fMRI paradigm. Another disadvantage is that the patients must lie motionless in the scanner during image acquisition, making this technique less suitable for children and other special populations.

One of the limitations of the previous fMRI studies in language mapping of patients with epilepsy has been their small sample sizes, resulting in low statistical power. While the largest study enrolled over 100 patients with epilepsy [19], even for a study with this size, it is difficult to distribute the subjects uniformly over the different subgroups of epilepsy. For example, some studies may have more patients with typical dominance for language than patients with atypical dominance for language. Systematic review with meta-analysis is one statistical technique that may overcome the small sample size problem faced by many clinical studies. Meta-analysis combines the results of several studies that address a set of related research hypotheses, with the general aim being to more powerfully estimate the true “effect size” as opposed to a smaller “effect size” derived in a single study under a given single set of assumptions and conditions. One example is a meta-analysis of English speaking healthy adult subjects performed by Vigneau et al. [20], who selected 126 studies among 260 published articles from 1992 to 2004. They separated the contrasts into three groups phonology, semantics, and sentence processing, and were able to find the relative clusters associated with each group. A similar study was performed by Medina et al. [21] who combined several related studies to investigate the role of fMRI to replace the Wada and ESM tests for epilepsy surgery using the Bayesian analysis approach. They concluded that the use of fMRI increased the final posttest probabilities of hemispheric dominance for language in patients with epilepsy.

## 4. Role of fMRI in Localization of Language Function

### 4.1. Intraoperative Localization of Language Functions by Electrocortical Stimulation (ESM) Technique

**Figure 4:**
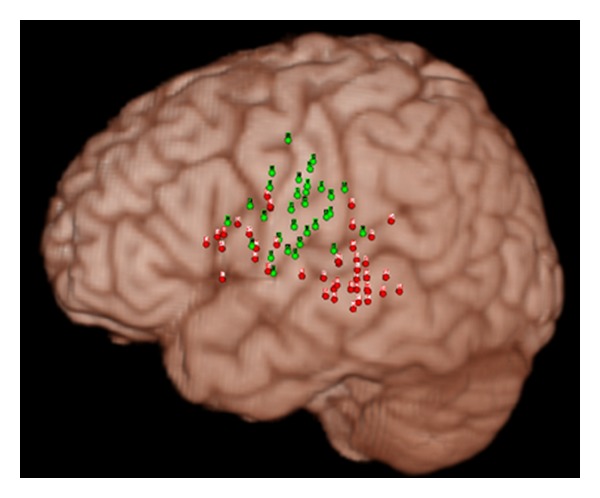
Electrocortical stimulation (ESM) sites from 13 patients with TLE of the left temporal lobe origin that are spatially normalized to MNI space and fused with MNI single subject brain.

ESM is a common routine clinical tool to localized language functions. This procedure involves direct application of electrical currents to the cerebral cortex of the awake patient, which either produces a response (e.g., movement) or disrupts function (e.g., speech arrest). ESM is believed to directly identify cortex essential for a specific task and can be performed extraoperatively in patients with implanted electrodes or intraoperatively with the cerebral cortex exposed at surgery. ESM is the “gold standard” tool for localization of language function during TLY.  Figure 4 demonstrates the ESM stimulation sites from 13 TLE patients who underwent surgery, normalized to MNI space [22], and fused with the MNI “Colin 27” single subject brain. The red and green dots represent the critical language and somatosensory sites, respectively. The red dots concentrated around the inferior frontal gyrus in the frontal lobe represent Broca's areas, while the cluster of red dots situated near the superior temporal gyrus represent Wernike's area.

Although it is widely accepted as the most reliable method of localizing language functions in the brain, ESM has several major drawbacks, including invasiveness, low spatial resolution due to limited sampling of the exposed cortex intraoperatively, the potential to induce epileptic after-discharges, as well as prolonging the operating time. Moreover, this technique is not always feasible in clinical practice, since it requires full collaboration of the patient as well as the clinical expertise of the surgical team [23]. In contrast to ESM, fMRI offers several appealing features for language localization, including noninvasiveness, preoperative data that can be employed to guide surgery and lead to reduced operating time, and superior spatial and temporal resolution for whole brain analysis. Accordingly, some studies have investigated the utility of fMRI to replace ESM for preoperative function localization in TLE surgical planning [24–29].

### 4.2. Comparison of fMRI to ESM for Localization of Language Functions

One of the first studies that compared fMRI and ESM included 28 patients, of whom 22 had epilepsy [24]. Two language tasks, number counting and word generation, which consistently activated mostly the inferior frontal lobe, were employed in this study, with results indicating a correspondence within 2 cm between fMRI and ESM using the word generation task. Another early investigation to compare fMRI and ESM was performed by FitzGerald et al. [25], who employed an array of five fMRI language tasks using both auditory and visual input to probe the language areas of 13 patients (one with epilepsy and eight with brain tumors). The sensitivity and specificity of fMRI for identifying language regions were calculated based on the ESM results. FitzGerald et al. concluded that the combination of language tasks was more effective than any single task and that fMRI was sufficiently sensitive to localize the language areas in these patients. Schlosser et al. [26] studied 33 patients with TLE, brain tumour, and AVM, using a single auditory comprehension task during the course of the study. They showed in 23 patients a consistent fMRI activation that was similar to that observed in healthy control subjects. Because in some patients the lesions were remote from the language areas, only 16 received intraoperative ESM for language localization. While four of their five TLE patients had a successful matching between fMRI and ESM, their correlation studies were rather qualitative and not discussed in detail.

Although some fMRI language paradigms have been developed and shown to activate language networks consistently in the normal population, it is expected that neurological disorders could affect the organization of the network and make these paradigms less effective in this population. Carpentier et al. [27] conducted a study in which a control group was compared with the diseased cohort to determine how the neurologically impaired group performed relative to the normals. While they found that their language tasks (visual and auditory comprehension) were able to successfully activate both Broca's and Wernike's areas in the healthy control group, the same array of language tasks resulted in lower activation and greater bilateral representations in the diseased group. Nevertheless, the concordance between fMRI and ESM was 100% (15 positive language sites identified by ESM matched with fMRI activation within a 1 cm range).

While a single language task activated some aspects of language network to achieve a good correlation with ESM in some of the previous studies, it is unlikely that this limited approach could fully explore the complete language networks. Therefore, several studies have been conducted to investigate the utility of multiple tasks to increase the likelihood of concordance between ESM and fMRI. Pouratian et al. [28] employed a battery of linguistic tasks, including visual object naming, word generation, auditory responsive naming, visual responsive naming, and sentence comprehension to identify language areas of 10 patients with vascular malformation. They found fMRI to be very sensitive, but rather unspecific, in identifying which cortical areas are essential for language. Rutten et al. [23] employed a battery of language tasks (i.e., verb generation, picture naming, verb fluency, and sentence comprehension) in their study and found that a single task could not elicit all the critical language areas that are elicited by ESM. They demonstrated that despite high sensitivity, on average only 51% of fMRI sites were confirmed by ESM. In contrast, in 10 out of 11 patients, the absence of fMRI activity was 100% concordant with the absence of critical language areas in ESM. Roux et al. [29] performed a study in which naming and verb generation tasks were administered to a group of 14 brain tumour patients. They also found that by combining the two language tasks they were able to increase sensitivity to 59% and specificity to 97%. Eight of their patients underwent postoperative fMRI, but only three of them showed an agreement between preoperative fMRI, postoperative fMRI, and ESM.

In general, good but not complete agreement between fMRI and ESM can be achieved when using a combination of carefully designed language paradigms. The inconsistency between the two techniques can be due to several factors. First of all, while the choice of language paradigm is of outmost importance to effectively activate the language network, there is still no consensus as to what combination of language paradigms is the most suitable for language mapping in an fMRI study, and this topic remains an active area of research. Furthermore, the classical human language system model consists of two primary regions: the expressive Broca's area in the posterior inferior frontal lobe, and the receptive Wernicke's area in the posterior superior temporal lobe. It has been shown that certain language tasks can activate Broca's area more effectively than Wernike's region. Therefore, it is expected that the sensitivity of fMRI to map language areas, and the concordance between fMRI and ESM may be task and lobe dependent. However, most studies performed so far have not examined the concordance at the lobe level.

The choice of the significance threshold used in statistical analysis for fMRI is important. The significance threshold is used to define the extent of language activation from the fMRI studies. A very strict threshold would result in very poor correlation (high Type II error) while a relaxed threshold would produce false positive correlations (high Type I error). The selection of threshold has usually been subjective and based on the previous experiences in these studies, and this variation may be partially responsible for the different results obtained. In addition, using a single fixed threshold across multiple subjects does not take into account the variability of activated language areas among individuals [25, 30]. An alternative approach to this problem is to apply an adaptive threshold that, based on the physiological observation of the extent of the language area, confines the area to be around 1 to 2 cm^2^ on the cortical surface. While this approach makes the comparison more consistent for all patients, in practice this can be very laborious.

**Figure 5:**
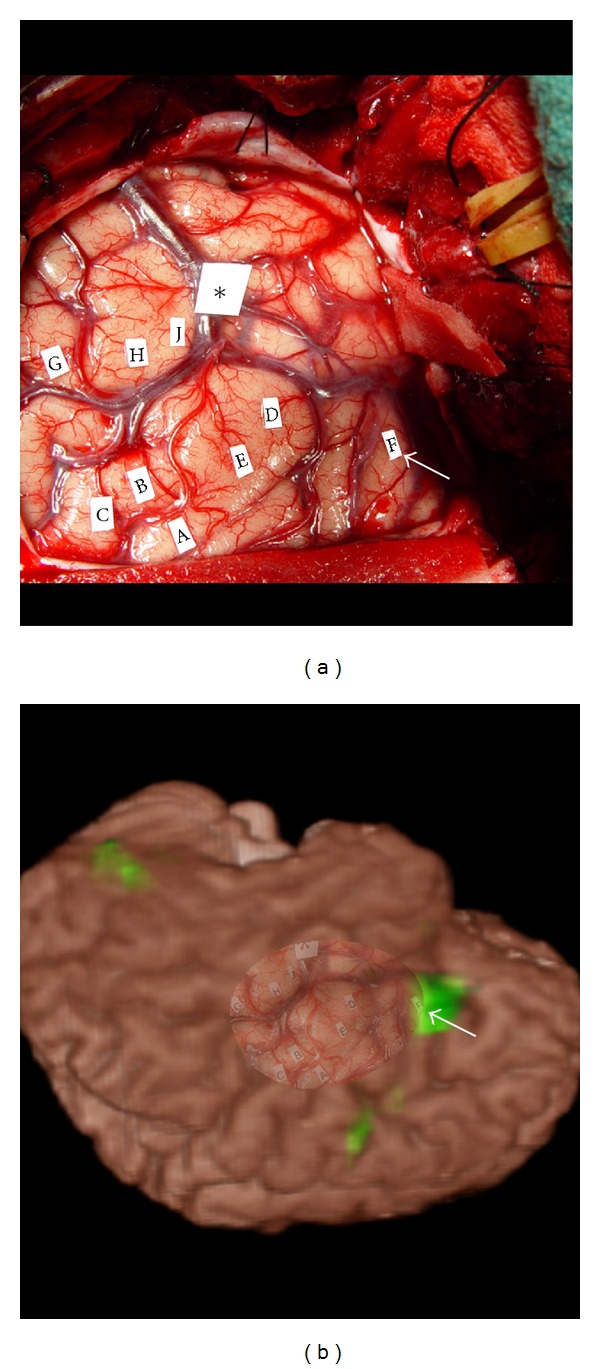
Overlay of intraoperative cortical images with fMRI for comparison. (a) Shows the intraoperative cortical photograph acquired just after ESM on the left temporal lobe. (b) Shows the overlay of this photograph onto volume-rendered anatomical MRI and fMRI. Arrows indicate where the label “F” is in the two images.

Finally, different registration approaches are used to align ESM and fMRI maps for comparison purpose. In several studies [23, 27], 2D ESM maps captured intraoperatively using a camera were manually correlated to the 2D fMRI maps and visually inspected, an approach that is considered to be subjective and qualitative. In contrast, other studies employed a relatively simple 2D-2D automatic rigid registration [24, 25, 28]. However, since 2D-2D registration does not account for the perspective effect in the 2D camera images, errors of several mm can remain in the registration. A third approach is to use a neuronavigation system [29]. However, such devices are not always available in many procedures, while simply imaging exposed cortical surface with a digital camera is a very common practice and requires less time to perform. An additional complicating effect is that the brain can deform up to 10 mm after opening the dura matter [31]. Without correction for this deformation, the accuracy of the reconstruction of the 3D coordinates of the stimulation sites is compromised by using the current navigation systems. This in turn degrades the fidelity of registration between ESM and fMRI. Figure 5 demonstrates our approach, which employs automatic 2D to 3D projective registration to fuse an interoperative cortical photographic image onto an MR brain image that incorporates a preoperative fMRI activation map [32]. In, Figure 5(a) the label “F” is a speech arrest site elicited by ESM that corresponds well to the peak of fMRI activation in the frontal lobe as shown in Figure 5(b).

## 5. Role of fMRI to Study Brain Plasticity for Language after TLY

Another area in which fMRI can play a role in the treatment of TLE is to study the language plasticity and reorganization before and after surgery. Previous fMRI studies have shown that ATL had a differential impact on the language functions of the left and right TLE patients [33]. Wong et al. [34] studied a group of 24 TLE patients who underwent ATL procedures to examine the impact of ATL on the cortical organization of language processing, using a verb generation task on both left and right TLE patients to compare their preoperative and postoperative fMRI response. After the surgery, the right TLE patients activated the same cortical network as before the surgery, while the left TLE patients elicited less activation. A subtraction analysis between the preoperative and postoperative BOLD response showed that the right inferior frontal gyri (IFG) and the left middle frontal gyri (MFG) were less activated after the surgery in the left TLE patients. The attenuated correlation between the language scores and the postoperative BOLD response within the IFG and MFG in both patient groups indicated cortical reorganization after the ATL. These findings suggest that the cortical organization of language processing is affected differently by the left and right TLE and is subsequently reorganized after ATL. Another finding of this study is that the right TLE patients shifted the correlation between their language scores and BOLD signals from the typical language areas (i.e., IFG and MFG) to the anterior cingulate cortex (ACC) after ATL. 

## 6. Conclusions

fMRI is a noninvasive technique that has replaced the invasive tests for presurgical assessment of the language network in some epilepsy centers. Generally, good concordance exists between fMRI and Wada or fMRI and ESM. However, there are still some requirements that fMRI must meet to enable its wider utility in the clinical practice for language mapping. First, a highly effective language paradigm or a battery of paradigms needs to be developed that can be administered to the patients in a clinically feasible time. Second, a robust statistical analysis methodology that removes individual biases (in selecting threshold levels, e.g.) must be developed. Finally, because the population being studied is, in general, atypical with respect to language localization, a high predictive power is required for critical language areas in the brain. These issues are currently being addressed one by one. With the availability of higher field strength MRIs, faster imaging sequences, better study paradigms, and improved postprocessing tools, the clinical applications of fMRI in epilepsy will only grow.
